# The Impact of Body Mass Index in Patients with Severe Burn Injury

**DOI:** 10.3390/ebj3030037

**Published:** 2022-08-22

**Authors:** Aline C. V. Walger, Lucienne T. Q. Cardoso, Marcos T. Tanita, Tiemi Matsuo, Alexandre J. F. Carrilho, Cintia M. C. Grion

**Affiliations:** 1 Londrina State University, Londrina 86038-350, Brazil; 2 Internal Medicine Department, Londrina State University, Londrina 86038-350, Brazil; 3 University Hospital, Londrina State University, Londrina 86038-350, Brazil; 4 Statistics Department, Londrina State University, Londrina 86038-350, Brazil

**Keywords:** obesity, burns, intensive care, body mass index, length of stay, hospital mortality

## Abstract

This study evaluated the association of body mass index (BMI) with mortality, length of stay in the intensive care unit (ICU), and length of hospital stay in major burn patients. It was a retrospective cohort study that was originally conducted from January 2017 to January 2020 and that used data from patients admitted to the intensive care unit for burns at a university hospital. The patients were divided into groups for the purposes of comparing relevant variables according to their BMI. We evaluated 288 patients: 52.8% were classified as eutrophic, 33.7% were classified as overweight, and 13.5% were classified as obese. The median length of stay in the ICU was 11 days for all patients, 9 days for eutrophic patients, 13 days for overweight patients, and 16 days for obese patients (*p* = 0.004). In the multivariate analysis, age (HR = 1.026; *p* < 0.001), total body surface area (HR = 1.047; *p* < 0.001), and the presence of inhalation injury (HR = 1.658; *p* = 0.026) were associated with mortality. Obesity was not associated with higher hospital mortality in this sample of burn patients. The length of stay in the ICU was longer among obese patients. Age, burned body surface, and the presence of inhalation injury were the major determinants of death in these patients.

## 1. Introduction

Obesity is a multifactorial disease, and its prevalence varies according to the country and period studied. The worldwide prevalence of overweight and obesity has doubled since 1980, to the point where nearly one-third of the world’s population is now classified as overweight or obese [1,2,3,4]. The method used by the World Health Organization to diagnose obesity is the body mass index (BMI), which is obtained by dividing the weight and the height of a patient squared. According to the World Health Organization, through BMI, patients are classified into five groups: BMI less than 18.5 kg/m^2^, low weight; BMI 18.5 to 24.9 kg/m^2^, normal weight; BMI 25 to 29.9 kg/m^2^, overweight; BMI 30 to 34.9 kg/m^2^, obesity grade 1; BMI 35 to 39.9 kg/m^2^ obesity grade 2; and BMI above 40 kg/m^2^, obesity grade 3 [2].

The impact of obesity on morbidity and mortality in the general population has been widely studied, and several studies have reported the association with cardiovascular disease, depression, diabetes mellitus, and even cancer [5,6,7]. As obese people represent an important percentage of the general population, many of the patients admitted to intensive care units also have this condition, accounting for 25% of the patients admitted to ICUs [8]. Some studies from the last decade have shown that obese or overweight patients in certain clinical situations may have lower mortality than normal weight patients and especially low weight patients, a fact that is currently called the “obesity paradox” [9,10,11].

The obesity paradox was first reported by Fleischmann et al. in 1999. That study evaluated the influence of overweight on mortality and length of hospital stay using 1346 hemodialysis patients in the state of Mississippi in the United States [12]. When compared to eutrophic patients, overweight and obese patients had lower mortality after 1 year of follow-up.

Other studies have evaluated the impact of BMI in critically ill patients, and unexpectedly, obesity was also related to a reduction in mortality in ICU patients for some specific diseases, such as sepsis. In 2016, Nguyen and collaborators published a cohort study using data collected from the Nationwide Inpatient Sample 2011 [13]. Mortality was reduced by 16% in obese patients; however, the average length of hospital stay and the cost of hospitalization were higher. These results were confirmed by Wang and collaborators in a meta-analysis in 2017 that evaluated the impact of overweight (BMI 25 to 29.9 kg/m^2^), obesity (BMI 30 to 39.9 kg/m^2^), and obesity grade 3 (BMI ≥ 40 kg/m^2^) on the outcomes of patients with sepsis [14].

The obesity paradox has also been reported in studies of patients with acute respiratory distress syndrome (ARDS) [15,16]. In 2016, Zhi et al. published a meta-analysis on the subject; when comparing normal weight patients with obese patients, obesity was associated with an increased incidence of ARDS [15]; however, the inverse was observed when evaluating mortality. When only analyzing patients who had already been diagnosed with ARDS, patients with BMI > 25 kg/m^2^ had a significant reduction in mortality.

The impact of obesity on surgical patients has also been evaluated by Hutagalung. After multivariate analysis, a BMI > 24.9 kg/m^2^ was independently associated with lower mortality [17]. The authors analyzed subgroups according to surgical specialty, with the impact of BMI presenting different behaviors in different surgical specialties. Patients with BMI ≥ 40 kg/m^2^ submitted to neurosurgery had a mortality rate 3× greater than that of eutrophic patients, demonstrating that perhaps these findings have a direct association with the underlying disease. In 2017, Rioz-Dias et al. studied the impact of obesity on the mortality of patients with soft tissue infections. The mortality of the studied population was 5.4%, and after adjusting for possible biases, obese patients (BMI > 30 kg/m^2^) showed a reduction in mortality when compared to eutrophic patients [18]. As demonstrated by all of these studies, BMI can have a paradoxically positive impact on the mortality of patients admitted to the ICU, but this impact can vary according to the disease that brought the patient to the ICU in the first place [19].

The impact of BMI on the mortality of critically burned patients is a topic that has been little studied to date. A study published in 2021 by Saadat et al. assessed whether increasing BMI to the Baux score could be useful for assessing mortality risk in burn patients. The authors found a difference in BMI between surviving patients and the patients who did not survive (28.2 vs. 23.0, *p* = 0.003) as well as an increase in hospital stay (24 vs. 5 days), suggesting that overweight could have a protective effect; however, because of the small sample of 56 patients, more studies are needed [20]. As burn patients have unique characteristics and because their mortality may be directly associated with burn characteristics such as burned body surface and the depth of the injury, in order to establish the relationship between mortality in major burn patients and BMI, it is necessary to study the impact of BMI on this group. The aim of this study was to verify the association of morbidity and mortality of major burn patients according to different BMI ranges.

## 2. Materials and Methods

### 2.1. Study Design and Patients

An observational study was carried out using data from patients admitted to the burn intensive care unit of a university hospital from January 2017 to January 2020. All burn patients over 14 years of age who were consecutively admitted to the intensive care burn unit during the study period were included. Patients with incomplete data were excluded from the study.

### 2.2. Measurements

The general data collected for all admissions to the burn treatment center were age, sex, weight, height, date of admission to the hospital and ICU, diagnosis on admission to the ICU, date of discharge from the ICU and hospital, and outcome at discharge from the ICU and hospital.

Data on the burn injury, such as the total body surface area, depth of the burn, and presence of inhalation injury, were recorded in addition to the etiological agent of the burn and type of accident that led to the injury. The abbreviated burn severity index (ABSI) was calculated using burn, age, and sex data [21]. Inhalation injury was confirmed by bronchoscopy or direct laryngoscopy.

Anthropometric data were collected from information provided by the patients when they were conscious at admission. The physiotherapy team measured the heights of all of the admitted patients. If it was not possible to acquire information about weight from the patient, weight was registered as reported by family members. If weight information was unavailable, an estimated value determined by at least three health professionals who assisted the patient on the first day of hospitalization was used.

### 2.3. Ethics

The study was conducted in accordance with the Declaration of Helsinki, and the protocol was approved by the Ethics Committee under the project identification code 94329218.8.0000.5231, opinion number 2,855,371. Due to the design and purpose of the study, the signing of the informed consent form was waived.

### 2.4. Statistical Analysis

The results of the continuous variables are described as the median and interquartile range (ITQ) due to the non-normal distribution of data. The Shapiro–Wilk test was applied to analyze the data distribution. The Kruskal–Wallis test was used to compare the medians of the continuous variables. Data were described using simple and percentage frequencies for qualitative variables. Statistical associations between two qualitative variables were assessed using the chi-square test or Fisher’s exact test when appropriate. The association and quantification of the risks with the outcome (death) was estimated by Cox proportional hazards regression analysis and described as the hazard ratio (HR) and a 95% confidence interval (95% CI). All analyses were performed at the 95% confidence level, and the 5% significance level was used. Analyses were performed using SPSS Version 19.0 (Armonk, NY, USA: IBM Corp.).

## 3. Results

**Figure 1:**
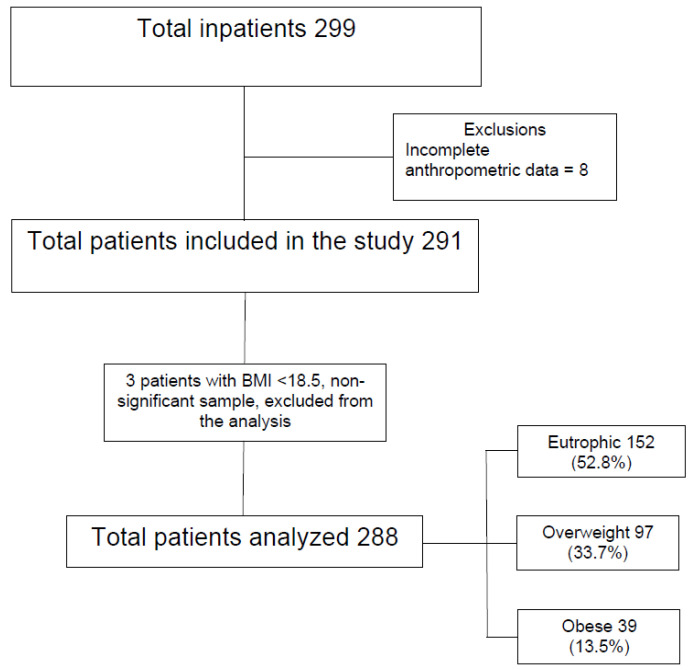
Flowchart of patients screened for study analysis.

Data were collected from 299 patients admitted to the ICU as burn patients during the study period. Subsequently, eight (2.6%) patients were excluded due to incomplete data in their medical records (referring to anthropometric data). Patients were then categorized according to their BMI: 3 patients (1.0%) were classified as low weight, 152 (52.8%) were classified as eutrophic, 97 (33.7%) were classified as overweight, 26 (9.0%) were classified as grade 1 obesity, 11 (3.9%) were classified as grade 2 obesity, and 2 (0.7%) were classified as grade 3 obesity (Figure 1). Due to the small number of patients with grade 2 and 3 obesity, patients with BMI ≥ 30 kg/m^2^ were analyzed together to comprise the group of patients with obesity, totaling 39 patients and representing 13.5% of the sample. The low-weight group was excluded from the statistical analysis due to the insufficient sample size. In most cases, burns were associated with domestic accidents, 55.2%, and work accidents, 24.3%. Fire (8.0%), attempted suicide (7.0%), attempted murder (4.0%), and car accidents (2.0%) were less frequent causes. The majority of injuries were classified as thermal injury burns, and alcohol was the main agent of this type of burn, being the cause in 41.0% patients.

Table 1 describes the main characteristics of the sample of hospitalized patients separated by BMI categories. The median age of the patients included in the study was 40.4 years old, and there was no significant difference between groups (*p* = 0.091). The prevalence of male patients was 67%. The most frequent comorbidities were hypertension (13.2%), diabetes (5.2%), smoking (14.2%), and alcoholism or drug addiction (13.9%). The median burned body surface was 22.0%, and 20.1% of patients had inhalation injury. It was possible to observe a gradually higher prevalence of arterial hypertension (*p* = 0.001) and diabetes (*p* = 0.029) in overweight and obese patients compared to eutrophic patients.

The overall mortality was 33.3%, and there was no difference between groups (*p* = 0.501). The mortality of eutrophic patients was 30.3%, the mortality of overweight patients was 37.1% (HR = 1.36, 95%CI: 0.794–2.329; *p* = 0.263), and the mortality of obese patients was 35.9% (HR = 1.29, 95%CI: 0.616–2.705; *p* = 0.500). The median length of stay in the ICU was 11 days for all patients, and this was similar between survivors (10 days) and nonsurvivors (11 days, *p* = 0.349). There was a gradual increase when the eutrophic patients were compared to the overweight and obese patients. The median length of stay was 9 days for eutrophic patients, 13 days for overweight patients, and 16 days for obese patients (*p* = 0.004). When analyzing the length of stay in the hospital, there was no difference between the groups (*p* = 0.176) (Table 2).

In the Cox proportional hazards model, the variables associated with in-hospital mortality were age (*p* < 0.001), total body surface area (*p* < 0.001), and the presence of inhalation injury (*p* = 0.026). According to the results of this model, each year of age increased mortality by 2.6%; each 1% of burned surface area increased mortality by 4.7%; and the presence of inhalation injury increased mortality by 65.8%. The variable of obesity did not have an impact on mortality (HR = 0.648, 95%CI: 0.350–1.210; *p* = 0.168) (Table 3).

## 4. Discussion

The present study evaluated the impact of obesity on the hospital mortality of burn patients admitted to an intensive care unit. In this group of patients, BMI did not have a protective effect on the outcome of death but was associated with a longer ICU stay.

The number of low-weight patients was small in our study, and therefore, the impact of this BMI class on the studied outcomes could not be evaluated. The percentage of overweight and obese patients, although significant, was lower than the percentage reported by other national statistics [3]. Due to the small number of patients in each obesity category, these groups could not be evaluated separately, which may have hampered data analysis, as in some publications, the benefit of the obesity paradox is more evident in patients with grade 1 and 2 obesity, with worsening mortality in patients with grade 3 obesity in some cases [11,12,13,14,15,16,17,18,19].

As for the profile of the patients, the present study aimed to assess the impact of obesity on severely burned patients needing ICU care and to determine whose mortality was higher. Overall, mortality was 33%, with a median ABSI of 6.0. There was no difference between groups regarding age, sex, burned body surface, or inhalation injury. However, overweight and obese patients had a higher incidence of hypertension and diabetes, which can be explained as obesity being a risk factor for the development of these diseases. Many patients were also smokers, alcoholics, or drug addicts, factors that were related to the accident’s mechanism of occurrence.

Initially, the mortality was compared between groups according to BMI, and no significant difference was observed. On the other hand, when the length of stay in the ICU was evaluated, overweight and obese patients had a gradual increase in their length of stay in the ICU. This may be explained by several factors that may delay discharge from the ICU, such as the presence of wound infections and problematic healing, the difficulty of monitoring this population, and the need to perform extensive dressing changes. In the current study, the duration of mechanical ventilation was not quantified, so this aspect could not be described; however, in another study of patients hospitalized in the same burn treatment center that evaluated the risk factors for ARDS in burn patients, the median mechanical ventilation time was 15 days, and BMI was not a risk factor for the development of ARDS [22]. Despite the difference in the length of stay in the ICU, when the length of stay in the hospital was evaluated, there was no difference between the groups. That is, the overweight and obese patients remained r in the ICU proportionally longer, but they did not need longer periods of hospital internment.

Regression analysis confirmed the absence of the obesity paradox in the present study, while intrinsic burn factors could have had an impact on the mortality outcomes. The main determinant of mortality, as expected, was the total body surface area and age. Unlike sepsis and ARDS, where the obesity paradox seems to be present [13,14,15,16], in major burn patients, the pro-inflammatory state of the obese patient and their greater metabolic reserves did not provide them with greater protection in a severe burn situation. However, an important limitation of the present study is the sample size of underweight and obese patients. So, for definitive conclusions on whether the obesity paradox exists in severe burned patients or not, more studies are needed.

Assessing the weight of a burn patient after starting volume expansion is not simple; in some patients, it was possible to obtain the patient’s anthropometric data from the patient themself, but at the time of admission to the ICU, some patients were already unconscious and, therefore, the weight had to be the estimated by the nursing team, which could have led to a measurement bias. However, the lack of precision of the anthropometric data that were collected and the possible biases associated with those data are not a problem exclusive to this study: other studies using large databases also present this limitation [16]. Another limitation of the study is the fact that it is a single-center study that represented a relatively small sample, and the results need to be clarified by other multicenter studies or meta-analyses.

## 5. Conclusions

In conclusion, this study could not detect an impact of obesity on hospital mortality in severe burn patients admitted to an intensive care unit. However, our data suggest that obese burn patients have a longer length of ICU stay as a secondary finding. Age, total body surface area, and the presence of inhalation injury were the major determinants of death in these patients, as demonstrated in previous studies.

## Figures and Tables

**Table 1:** Main characteristics of patients according to body mass index.

	BMI 18.5–24.9	BMI 25.0–29.9	BMI ≥30.0	*p*-Value
Age: median (q1–q2)	36.3 (24.9–55.4)	42.7 (30.8–52.9)	43.9 (32.8–59.7)	0.091
Male sex %	102 (67%)	65 (67%)	26 (66.7%)	0.999
TBSA: median (q1–q2)	20.0 (12.6–29.8)	25.0 (14.0–37.5)	23.5 (10.0–32.0)	0.243
Inhalation injury	27 (17.8%)	23 (23.7%)	8 (20.5%)	0.520
ABSI: median (q1–q2)	6.0 (5.0–7.0)	7.0 (5.0–8.0)	6.0 (5.0–7.0)	0.218
Hypertension	13 (8.6%)	13 (13.4%)	12 (30.8%)	0.001
Diabetes	3 (2%)	8 (8.2%)	4 (10.3%)	0.029
Smoking	18 (11.8%)	16 (16.5%)	7 (17.9%)	0.459
Alcoholism and/or Drug addiction	25 (16.4%)	12 (12.4%)	3 (7.7%)	0.321

BMI = body mass index, TBSA = total body surface area, ABSI = abbreviated burn scoring index; SD = standard deviation.

**Table 2:** Outcomes of burn patients according to body mass index.

		Body Mass Index Categories			
All Patients		BMI 18.5–24.9	BMI 25.0–29.9	BMI ≥ 30.0	*p*-Value
Length of ICU stay (days): median (ITQ)	11.0 (5.0–19.0)	9.0 (4.0–16.8)	13.0 (5.5–20.0)	16.0 (7.0–29.0)	*p* = 0.004 *
Length of hospital stay (days): median (ITQ)	19.0 (11.0–32.0)	18.0 (10.0–29.0)	21.0 (13.0–30.0)	22.0 (12.0–39.0)	*p* = 0.176 *
Mortality	96 (33.3%)	46 (30.3%)	36 (37.1%)	14 (35.9%)	*p* = 0.501 **

Legend: * Kruskal–Wallis test; ** Chi-square test.

**Table 3:** Cox proportional hazards model in relation to risk factors for death in patients admitted to the burn ICU.

Variables	Hazard Ratio	95%CI	*p*-Value
Body Mass Index			
Overweight	0.753	0.478–1.188	0.223
Obesity	0.648	0.350–1.201	0.168
Age	1.026	1.014–1.038	<0.001
Males	1.239	0.793–1.935	0.346
Total body surface area	1.047	1.036–1.058	<0.001
Inhalation injury	1.658	1.061–2.590	0.026

## Data Availability

The data presented in this study are available from the corresponding author upon request.
